# Measurements of Surgical Volume in Low- and Middle-Income Countries, a Systematic Review

**DOI:** 10.5334/aogh.4251

**Published:** 2023-10-11

**Authors:** Ifeoluwa Shoyombo, Abraham Genetu, Lye-Yeng Wong, Muhammed Elhadi, Eric Twizeyimana, Grace Paidamoyo Gwini, Rutikanga William, Timothy Hall, Halimah Khalil, Siva Nyanamani Sandrasagran, Monica Langer

**Affiliations:** 1 Johns Hopkins University, Department of Surgery, 1800 Orleans St, Baltimore MD 21287, US; 2 Addis Ababa University, Department of Surgery, Cardiothoracic Unit, Zambia Street, Addis Ababa, ET; 3 Stanford Hospital, Department of Cardiothoracic Surgery, 870 Quarry Ext Rd, Stanford, CA 94304, US; 4 Faculty of Medicine, University of Tripoli, Tripoli, LY; 5 Furnaj, University Road, Tripoli, Libya, 13275, Tripoli, LY; 6 Center for Equity in Global Surgery, University of Global Health Equity, Kigali Heights, Plot 772, KG 7 Ave, 5th Floor, PO Box 6955, Kigali, RW; 7 School of Public Health, Faculty of Health Sciences, University of Cape Town, Observatory, 7925, ZA; 8 Kibungo Referral Hospital, RGMQ+QQ8, Kibungo, RW; 9 Waikato Hospital, Urology Department, 183 Pembroke Street, Hamilton 3204, NZ; 10 College of Medical and Dental Sciences, University of Birmingham, Birmingham, UK; 11 Mackay Base Hospital, 475 Bridge Road, West Mackay 4740 QLD, AU; 12 Lurie Children’s Hospital of Chicago, 225 E Chicago Ave, Chicago, IL 60611, US

**Keywords:** surgical volume, global surgery, low- and middle-income countries

## Abstract

**Background:**

Surgical volume is a surgical indicator that was described in the Lancet Commission on Global Surgery (LCoGS) and the World Bank World Development Indicators as an important metric for tracking the delivery of surgical care.

**Objectives:**

We aimed to characterize the reports on surgical volume (SV) in the existing literature by using a systematic review to assess studies that examine surgical procedures as a ratio of a population (procedures/100,000 population).

**Methods:**

The PRISMA guideline was employed in the systematic review of articles that addressed the measurement of SV in low- and middle-income countries (LMICs), with the primary outcome of surgical procedures/100,000 population.

**Findings:**

The search result consisted of 6,657 preliminary studies. Following the title and abstract screening, 6,464 articles were excluded, and the remaining 193 were included in the full text review. From the full text review of the 193, only 26 of these articles defined SV as the ratio of number of procedures per population of the catchment/geographical area. The reported SV was a mean of 765, with an SD of 1260 operations per 100,000. The median SV was 180 (min = 0.900, max = 4470).

**Conclusion:**

Our findings support the LCoGS assessment of the gap in surgical care. The target for SV is 5000 per 100,000 population, compared to the average of 765 per 100,000 population as found in this review. The challenges for assessing surgical volume gaps are vast, including the nature of written records, which limits SV reports to an absolute number of procedures per year without a reference to the catchment population. For the purpose of tracking SV, we recommend using proxies that account for the capacity of facilities to deliver care regardless of the catchment population.

## Introduction

Surgical conditions account for 20%–35% of the global burden of disease, with 11% of global disability-adjusted life years (DALYs) attributable to surgical diseases [1]. The Lancet Commission on Global Surgery (LCoGS) identified that five billion people do not have access to safe, affordable surgical, obstetrics, trauma, and anesthesia (SOTA) care [2]. An astonishing nine out of ten people in low- and middle-income countries (LMICs) lack access to basic SOTA care. The nature of this issue is urgent in light of the third goal of the United Nation’s sustainable development goals and declaration in September 2015 to ensure healthy lives and promote well-being for all ages [3]. With this gap in surgical care, the strengthening of global systems and surgical scale-ups have been set as a priority [4]. This crucial need for a surgical scale-up has been foreshadowed by research and measurement sciences, which have been developed to track the progress of the capacity-building efforts that are necessary to increase surgical volume (SV) among the population of those who have limited access to SOTA care. Hence, the LCoGS proposed tracking several indicators, including access to surgery, surgical workforce, SV, perioperative mortality rate, and the catastrophic and impoverishing financial consequences of surgery. Each of these indicators provides targets for assessing improvements or stagnancies in the assessment of surgical capacity. The importance of these metrics is very significant, and as a result, have been incorporated into the World Bank World Development Indicators.

Surgical volume (SV) is a unique metric, as it measures met needs with a goal of addressing the 143 million additional operations per year that are needed to decrease the global burden of surgical diseases [2]. This metric qualifies the relationship of existing healthcare infrastructure and the surgical disease that is present in the catchment population of the hospital. It was originally defined by the LCoGS as the number of procedures done per 100,000 population. This definition, recently expanded on by global experts who convened in 2019, has been redefined as the “number of surgical procedures done in an operating theater using any form of anesthesia, per 100,000 population per year [5].” The broadening of this definition was redefined to address the varying definitions and breadth of surgical procedures, which are defined as an incision, excision, or manipulation of tissue using anesthesia in an operating theater, including day-cases but excluding surgical procedures outside of the operating room. Furthermore, the definition of anesthesia in the context of surgical procedures was unanimously defined as regional anesthesia, general anesthesia, or profound sedation that is necessary to control pain during a surgical procedure.

With an LCoGS target of 5000 procedures per 100,000 population, surgical volume is at the forefront of assessing whether surgical needs are adequately met. By 2030, the global SOTA community hopes to have reached the LCoGS—with a realization that to measure the progress toward the target, it is expected that at least 80% of countries report this indicator by 2020, and 100% by 2030. Yet, there are no reviews evaluating how this indicator is currently being used in LMICs, where the need for surgical scale-ups is the highest. The peculiar challenges of tracking surgical volume need to be highlighted to understand the feasibility of tracking surgical volume at different levels of care. The aim of this review is to understand the measurement of surgical volume in the existing literature. We aim to identify the challenges, alternatives, and solutions to surgical volume measurement that have been reported on and published in the literature, with a goal of providing insights and recommendations on improving the measurement of surgical volume. Our secondary aim is to assess whether any specific interventions have shown promising potential to significantly increase surgical volume.

## Methods

The study protocol was approved by the international prospective register for systematic reviews. Using the PRISMA guidelines, the relevant MeSH terms, including “surgical volume” and “low- and middle-income country” were searched in Global Health (EBSCO), LILACS (WHO), MEDLINE (Ovid), Scopus (Elsevier), and the Cochrane Library (Wiley). The inclusion criteria for the systematic review included surgical patients in LMICs (including Obstetrics/Gynecology and Trauma). Exclusion criteria included percutaneous (cardiac or vascular) intervention, endoscopic interventions, cosmetic surgery, short-term medical missions, elective abortion, vaginal births, robotic operations, ECMO, bariatric surgery, trauma sustained during a military engagement, and surgery performed in temporary combat hospitals, studies with total <5 patients. All types of studies were included in this review with a timeline of January 1990 to the present.

The primary outcome was the recording of studies measuring SV in LMICs, defined as the number of procedures done in an operating theater per 100,000 population per year, where the procedure is understood as the incision, excision, or manipulation of tissue that requires regional or general anesthesia, or profound sedation to control pain. Secondary outcomes were the proportion of studies evaluating SV variations as a result of an intervention in LMICs and the proportion of studies associating health insurance coverage and SV in LMICs.

Records were managed using Rayyan, an online software that allows reviewers to collaborate, while keeping them blinded to each other’s decisions. Data was extracted to a Google spreadsheet. A calibration exercise was conducted before the review to ensure that all reviewers understood the selection criteria. Titles and abstracts were screened and reviewed by two independent reviewers to identify studies that met the inclusion criteria. Disagreements on the eligibility of particular studies, based on their title or abstract, were resolved through discussion and adjudication by a third reviewer. The full text was obtained to aid decision-making when considered appropriate. The studies selected for full text review were read by trained data extractors to evaluate inclusion and exclusion criteria, with final oversight and resolution of disagreements by additional blinded reviewers. Data extraction was standardized and collected on a spreadsheet.

Data extracted include demographic information, methodology, intervention details, and outcomes. The disaggregation of surgical volume indicators has been described in the LCoGS and the Utsein report [2,5]. Hence, the data extraction included: basic study data including study population data/demographics; timing; surgical volumes; specifics of surgical procedures/type of anesthesia (if available); any interventions taken to increase SV; availability/proportion of the population with health insurance and whether this includes surgical care and specifically cesarean sections; generalizability; and interventions or adjustments to facilitate SV data collection. We used descriptive statistics to describe the results.

Risk of bias was assessed by two independent reviewers using the ROBINS checklist, with each study rated as having: low risk of bias, moderate risk of bias, serious risk of bias, critical risk of bias, or no information. Two reviewers determined the strength of the body of evidence using the Grading of Recommendations Assessment, Development, and Evaluation (GRADE) working group methodology. Certainty of evidence was classified as either high, moderate, low, or very low based on the scoring criteria.

## Results

**Figure 1:**
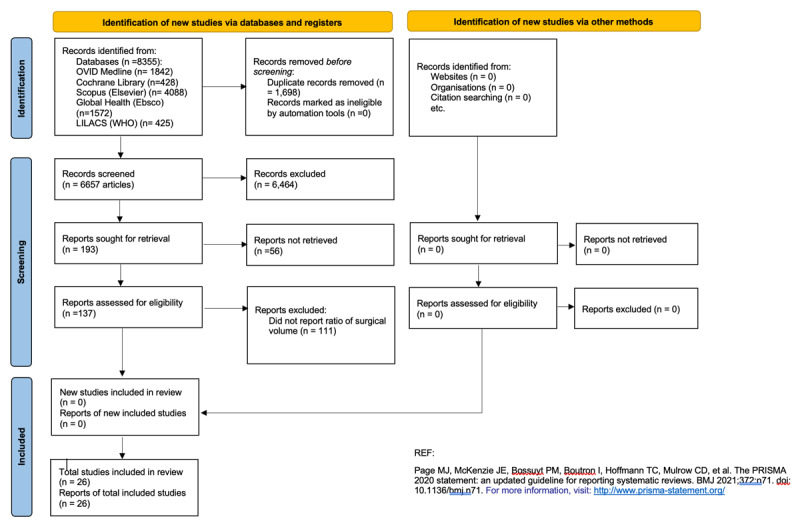
PRISMA 2020 flow diagram for updated systematic reviews which included searches of databases, registers and other sources.

The search result consisted of 6,657 preliminary studies. Following the title and abstract screening, 6,464 studies were excluded, and the remaining 193 were included in the full text review. From the full text review, we excluded 56 and included 137 studies. The PRISMA flow chart is shown in Figure 1. Only 26 of the 137 articles defined SV as the ratio of number of procedures per population of the catchment/geographical area. Table 1 lists the 26 included studies and provides basic data, such as title, first author, journal, year, and the country of study.

**Table 1:** List of 26 included articles that report surgical volume.


FIRST AUTHOR	TITLE OF PAPER	YEAR	JOURNAL NAME	COUNTRY OF STUDY

Martins, Monica	Access and effectiveness inpatient care indicators and economic crisis: analysis based on the Brazilian Unified Health System data, Brazil and southern Brazil states, 2009–2018 [6]	2019	*Cicencia e Saude Coletiva*	Brazil

Albutt, Katherine	Access to safe, timely, and affordable surgical care in Uganda: a stratified randomized evaluation of nationwide public sector surgical capacity and core surgical indicators [7]	2018	*World Journal of Surgery*	Uganda

Caligaris, L	Analyses of cataract surgery performed by the Unified Health System in Brazil, 2006–2007 [8]	2011	*Revista Panamericana de Salud Publica/Pan American Journal of Public Health*	Brazil

Sameen, Siddiqi	Are rural hospitals in Pakistan responding to the global surgery movement? An analysis of the gaps, challenges and opportunities [9]	2020	*World Journal of Surgery*	Pakistan

Bjerring, Anders W	Assessing cesarean section and inguinal hernia repair as proxy indicators of the total number of surgeries performed in Sierra Leone in 2012 [10]	2015	*Surgery*	Sierra Leone

Massenburg, Benjamin	Assessing the Brazilian surgical system with six surgical indicators: a descriptive and modelling study [11]	2017	*BMJ Global Health*	Brazil

Liwattananon, Chulaporn	Association between a centrally reimbursed fee schedule policy and access to cataract surgery in the Universal Coverage Scheme in Thailand [12]	2018	*JAMA Ophthalmology*	Thailand

Gyedu, Adam	Benchmarking global trauma care: Defining the unmet need for trauma surgery in Ghana [13]	2020	*Journal of Surgical Research*	Ghana

Pawiroredjo, Jerrel	The cataract situation in Suriname: An effective intervention programme to increase the cataract surgical rate in a developing country [14]	2017	*BMJ Opthalmology*	Suriname

Habtamu, Esmael	Cataract surgery in Southern Ethiopia: distribution, rates and determinants of service provision [15]	2013	*BMC Health Services Research*	Ethiopia

Chen, Xiaofan	The effect of health insurance reform on the number of cataract surgeries in Chongqing, China [16]	2011	*BMC Health Services Research*	China

Fenton, Paul	The epidemiology of district surgery in Malawi: a two year study of surgical rates and indices in rural Africa [17]	2011	*East and Central African Journal of Surgery*	Malawi

Jamison, Aron	Establishing a pediatric ophthalmology service in Malawi: developments in childhood cataract surgery [18]	2019	*Middle East African Journal of Opthalmology*	Malawi

Melo, Gusatvo	Incidence of endophthalmitis after cataract surgery (2002–2008) at a Brazilian university-hospital [19]	2010	*Arquivos Brasileiros de Oftalmologia*	Brazil

Burgos, C	The met needs for pediatric surgical conditions in Sierra Leone: estimating the gap [20]	2018	*World Journal of Surgery*	Sierra Leone

Concepcion, T L	Provision of surgical care for children across Somaliland: challenges and policy guidance [21]	2019	*World Journal of Surgery*	Somaliland

Nordberg, E	Rates of major surgery by age and sex in a rural district in Kenya [22]	1996	*Annals of Tropical Medicine & Parasitology*	Kenya

Chichom-Mefire, A	A retrospective one-year estimation of the volume and nature of surgical and anaesthetic services delivered to the populations of the Fako division of the South-West Region of Cameroon: an urgent call for action [23]	2017	*World Journal of Surgery*	Cameroon

Malavolta, E A	Rotator cuff repair in the Brazilian Unified Health System: Brazilian trends from 2003 to 2015 [24]	2017	*Brazilian Journal of Orthopedics*	Brazil

Anderson, G A	Surgical volume and postoperative mortality rate at a referral hospital in Western Uganda: measuring the Lancet Commission on Global Surgery indicators in low-resource settings [25]	2017	*Surgery*	Uganda

Nabembezi, J S	Surgical output in Kibaale District, Uganda [26]	2001	*East African Medical Journal*	Uganda

Bruno, E	An evaluation of preparedness, delivery and impact of surgical and anesthesia care in Madagascar: a framework for a national surgical plan [27]	2017	*World Journal of Surgery*	Madagascar

Zhu, M	Four-year analysis of cataract surgery rates in Shanghai, China: a retrospective cross-sectional study [28]	2014	*BMC Ophthalmology*	China

Weiser, T G	Size and distribution of the global volume of surgery in 2012 [29]	2016	*Bulletin of the World Health Organization*	66 WHO Member states

Reshamwalla, S	Snapshot of surgical activity in rural Ethiopia: is enough being done? [30]	2012	*World Journal of Surgery*	Ethiopia

Henry, J A	Surgical and anaesthetic capacity of hospitals in Malawi: key insights [31]	2015	*Health Policy & Planning*	Malawi


**Figure 2:**
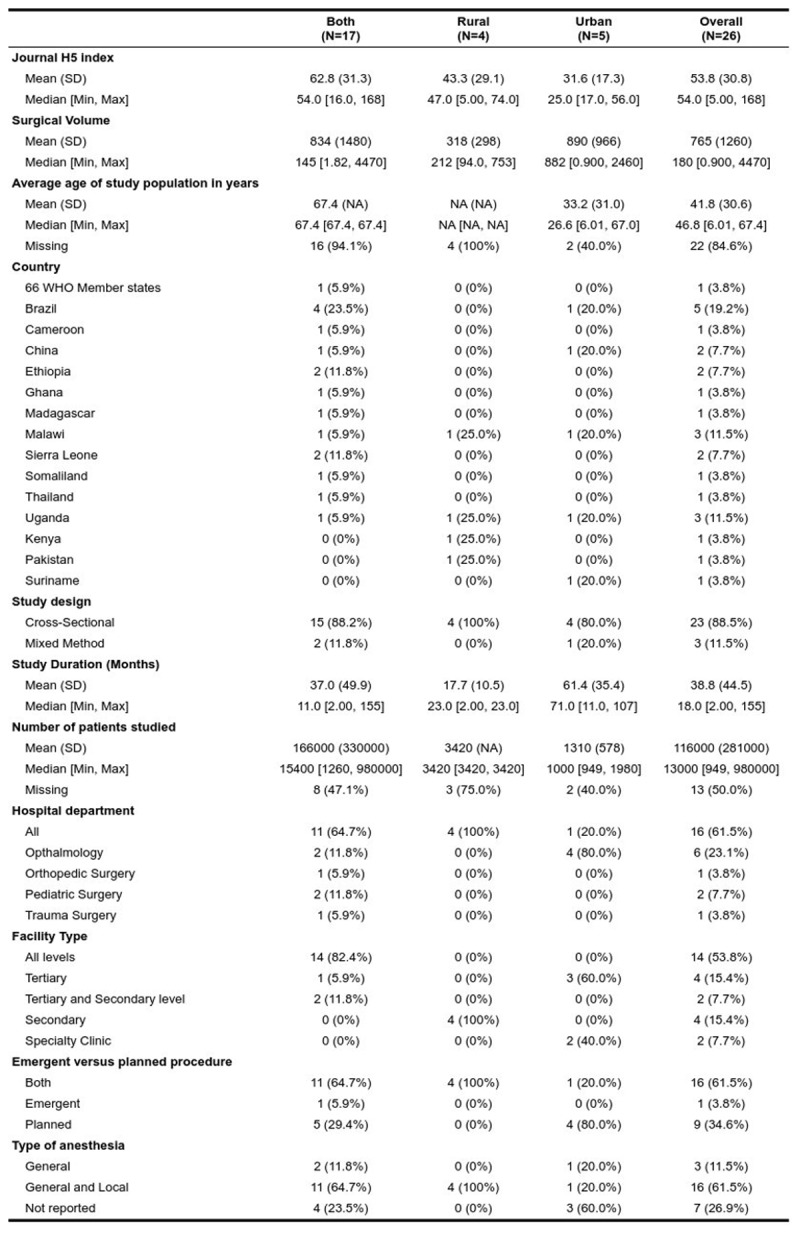
26 included studies based on the study settings of urban, rural, or both.

We stratified the 26 included studies based on the study settings of urban, rural, or both (Figure 2). Most of the articles assessed the SV in both rural and urban facilities. Four studies examined rural hospitals, and five studies examined urban hospitals alone. The average journal H5 index was 53.8, with a median of 54 (IQR 5–168). The mean reported surgical volume was 765 operations per 100,000 (SD 1260). The median SV was 180 (IQR 0.900–4,470). Age was not consistently reported. In fact, 22 (84.6%) articles did not report on age at all. However, of the four articles that reported age and range, the mean was 41.8 (SD 30.6), and the median age was 46.8 (IQR 6.01–67.4).

The most represented country was Brazil, with a frequency of five studies, followed by Malawi and Uganda, which had a frequency of three studies each. Twenty-three (88.5%) of the studies were done with a cross-sectional method. Three of the studies included a prospective cohort mixed with estimate modeling methods. Average study duration was 38.8 months (SD 44.5). The mean number of patients studied of the included studies was 116,000 (SD 281,000). However, there were 13 articles with missing entries.

Most of the studies examined all the surgical departments in a hospital (n = 16), however, there was a high representation of the ophthalmology department (n = 6) among the subspecialty departments, with the most studied diagnosis being cataracts. Most of the studies examined primary, secondary, and tertiary hospitals. Four of the studies examined tertiary centers only, and four other studies examined secondary centers only. None of the studies assessed the surgical volume in primary facilities alone. Most of the studies assessed emergency and planned surgery simultaneously. Only one of the studies looked into emergency surgery alone, whereas nine studies assessed planned studies. The most common type of anesthesia was general anesthesia, which constituted 73% of the studies. However, seven articles did not explicitly state what type of anesthesia was involved in the study.

GRADE assessments were performed on the 26 studies, and the certainty of evidence was categorized as “low” or “very low” in all included studies. All the studies were observational or cross-sectional, which is generally assigned a “low” certainty of evidence at the start of the process. The studies were subsequently down rated due to high risks of bias and inconsistencies throughout. Indirectness, imprecision, and publication bias were also assessed but contributed less to the overall rating than the aforementioned two domains.

## Discussion

In this systematic review, we attempted to quantify SV in LMICs based on the available literature from the last three decades. Our findings highlight a large gap in both studying and reporting this important indicator, and the inconsistencies in the study definitions for SV greatly hamper the generalizability and aggregability of the data. As the most represented subspecialty group in the report, the ophthalmologic studies on surgical volume prove to be the most standardized across all the studies. The number of procedures was consistently reported by these articles as a count of operations over a period of time, as compared to the general population in a local or national region. For example, the cataract surgery rate that was assessed by Caligaris et al. reported the population denominator as the general population of the entire country of Brazil. From the counts of the number of cataract procedures and the population data, Caligaris et al., along with all of the ophthalmology articles included in this report, were able to calculate the rates of cataract surgery, which was reported as cataract surgery per million people per year.

Within the subspecialties, studies that addressed obstetrical cesarean sections alone were excluded (*n* = 9) from the review because they reported surgical volume as the absolute number of cesarean sections (CS) as a ratio of the total number of live births. The denominator variable, total live births, would need to be transformed to make it suitable as a catchment population variable. Total live births as a denominator variable is not a sustainable analysis for comparing CS capacity among hospitals; information on catchment population is very important in making inferences about facilities’ capacity to meet unmet obstetric needs. There are some transformations that could be calculated from total live births. The crude birth rate, which is the total number of births divided by the population multiplied by 1,000, would require a population estimate for the specified time period to make the transformation possible. This is attainable using world population data, as was done by Bruno et al., where the population estimates were calculated using population estimates on the world bank repository [27]. Nevertheless, a unique approach to assessing surgical volume was highlighted by Kushner et al., given that using the relative frequency of CS to total operations to calculate a percent c-section (%CS) at a hospital can serve as a proxy for measuring surgical capacity, which is an aspect of the aim of measuring SV [28]. In high-income countries, for example, %CS is about 2.6% of the total procedures [28,29]. Following a World Health Organization (WHO) survey of 40 district hospitals in Rwanda, Petroze et al. determined that hospitals with a high %CS were less likely to have capacity for blood banking, amputation, closed fracture repair, inhalation anesthesia, and chest tube insertion [29].

Most of the articles assessing surgical volume relied on hospital case logs and charts to determine the number of surgical procedures within the duration of the study. Even sophisticated assessment tools like the Surgical Assessment Tool (SAT), a tool developed by the Program in Global Surgery and Social Change and the WHO, relied on perioperative case logs to calculate the number of procedures within the catchment areas [30,31,32]. Nevertheless, the difficulty in assessing surgical case logs is demonstrated in the study by Henry et al. It explored data obtained by a visit to every district hospital over the course of a year and a manual review of operating theater logbooks [33]. The present means of obtaining case logs, which is intrinsically important for calculating surgical volume, is quite tedious. Although the LCoGS provided a valid rationale for the need to track surgical volume, there has been poor documentation, an absence of standardization, and difficult data collection and management, which makes it difficult to assess these metrics [34]. Anderson et al. assessed the validity of using logbooks versus patient charts [25]. Retrieving files of patients from the medical records, staff sometimes took excessive amounts of time, sometimes up to several weeks. In addition, patient charts were found to disproportionately represent obstetric operations (68.3% versus 49.2%, *p* < 0.001) and to underrepresent charts from pediatric patients (3.7% versus 13.0%, *p* < 0.001) [25]. Logbooks, on the other hand, were found to be rapid, simple, accurate, and effective for determining surgical volume. There is a dire need to improve the maintenance of perioperative case logs that enable easier data collection and are electronically accessible. This would improve the capacity of hospitals in LMICs to perform accurate surgical volume calculations. Looking for charts retrospectively from medical records has proven to be low yield, and in a setting with already low work force density, tasking hospital staff with obtaining information from medical records would take away time from patient care and be counterproductive.

Ideally, a registry that can be used to maintain procedures, diagnoses, and other disaggregation variables to determine the numerator of SV would be very appropriate. Of all the countries that were included in this study, Brazil had the most representation compared to any other region. The reason for this is most likely because the Brazilian studies were able to easily use the Brazilian Unified Health System (SUS), developed by the Department of Public Health Care System (DATASUS), as the source of their data [32]. As a result, there was consistency in the metrics used by the researchers. DATASUS is managed and validated by the central government and the ministry of health. The availability of DATASUS made data incredibly accessible to researchers in order to assess metrics such as SV. This model of centralized data collection is important to achieve milestones in surgical volume tracking. Enabling these research capacities will improve the quality of data, which can be used to inform the progress of SV and compare progress with other hospitals in the LMIC sphere. Understandably, there would need to be infrastructure investment in equipment, training, and internet access to make these attainable, especially in the context of achieving increased surgical volume before the target year of 2030.

Insurance and universal health coverage plans that involve surgical care were shown to have a positive correlation. Several of the surgical data from the ophthalmological studies included in this study assessed the impact of health insurance on the increase of surgical volume. For example, in 2002, Thailand implemented universal health coverage for uninsured and medically indigent people. The effect of the policies was assessed by Limwattananon et al [12]. The intervention that was assessed involved a central reimbursement (CR) model, which involves retrospective reimbursement for selected medical and surgical procedures. However, in 2011, the National Health Security Office, a Universal Coverage Scheme administrative body in Thailand, introduced a dual-payment method with fixed schedules of payment for surgical procedures, including intraocular lens insertion and extraction. This policy was modified in 2013, whereby mobile clinics were barred from most regions. Overall, during the nine years of CR implementation, the national cataract surgery rate, which is a surgical volume measure, more than doubled, reaching 765.3 cases per 100,000 population from a baseline of 352 cases per 100,000 population. There was a lag between 2005 to 2008 following the introduction of the CR, which was followed by an increase of 64.9 cases/100,000 population per year. Following the barring of fixed fee and mobile services, the rate decreased to 34.8 cases per 100,000 population. In China, Zhu et al. assessed the cataract surgery rate during the implementation of cataract blindness prevention. This occurred in conjunction with a booming economy in 2006 that resulted in a GDP that was more than 10-fold that of India at the time. The collection of the data was made possible following the implementation of government policies in 2003, and it drove the recoding of cataract surgeries in a Cataract Operations Registration Form, which includes name, sex, home address, cataract classification, detailed ophthalmological exam information, cataract surgeon, surgical management, intraocular lens implantation, cost, and operative outcome. There was a requirement for all cataract surgery units to be completed before the fifth day of each month. The data was reported to Eye Disease Prevention Centers in 19 districts, and random inspections were performed by the Eye Disease Prevention and Treatment Center in order to review the quality of the data.

We acknowledge several limitations in our study, including the fact that the quality of surgeries was often not reported. Also, none of the research articles that met the inclusion criteria focused specifically on primary levels. Rather, tertiary, secondary, and specialty clinics were overrepresented. Yet, we believe that this article highlights the current gaps in defining and describing SV in LMICs and can provide a stepping stone for healthcare systems to intervene on a national level. As safe and quality surgical access is improved and organization systems such as perioperative registries are developed in LMICs, the literature on SV will hopefully be more abundant and help policymakers set attainable and objective goals for expanding essential surgical services to all.
